# The Relationship between Vitamin D Levels and Blood Glucose and Cholesterol Levels

**DOI:** 10.3390/clinpract14020032

**Published:** 2024-02-29

**Authors:** Eman Elsheikh, Abdulhakim Ibrahim Alabdullah, Sarah Saleh Al-Harbi, Amal Omar Alagha, Dhiyaa Hassan AlAhmed, Mazen Moraya Ali Alalmaee

**Affiliations:** 1 Internal Medicine Department, College of Medicine, King Faisal University, Alahsa 31982, Saudi Arabia; 2 Cardiovascular Department, College of Medicine, Tanta University Hospital, Tanta 31527, Egypt; 3 College of Medicine, King Faisal University, Alhasa 31982, Saudi Arabia; 219022852@student.kfu.edu.sa (A.I.A.); 220025375@student.kfu.edu.sa (D.H.A.); 218038106@student.kfu.edu.sa (M.M.A.A.); 4 Pharm.D., College of Pharmacy, Umm Al-Qura University, Makkah 21955, Saudi Arabia; s438005876@st.uqu.edu.sa (S.S.A.-H.); s438011107@st.uqu.edu.sa (A.O.A.)

**Keywords:** vitamin D, diabetes, dyslipidemia, metabolic syndrome, Saudi Arabia

## Abstract

Background: Vitamin D deficiency has reached epidemic proportions globally. Observational data link low vitamin D status to diabetes, dyslipidemia, and metabolic syndrome, but interventional trials on the effects of supplementation are limited. Objective: We investigated associations between serum 25-hydroxyvitamin D (25(OH)D) levels and metabolic markers in Saudi adults. Methods: This retrospective cross-sectional study analyzed the clinical records of 476 patients from Saudi Arabia, aged 15–78 years. According to 25(OH)D levels, participants were stratified as vitamin D-sufficient (≥30 ng/mL), -insufficient (21–29 ng/mL), or -deficient (≤20 ng/mL). The outcomes were diabetic status (fasting glucose, HbA1c) and lipid panel results. Results: Higher diabetes prevalence was significantly associated with lower 25(OH)D levels (10.1% in the sufficient group, 11.6% in the insufficient group, and 18.3% in the deficient group). Similarly, worse lipid profiles were associated with more severe hypovitaminosis D, including a total cholesterol level of ≥240 mg/dL (5.3% in participants with normal vitamin D levels vs. 18.9% in those with deficient levels) and LDL ≥ 160 mg/dL (6.9% in participants with normal vitamin D levels vs. 13.2% in those with deficient levels). Vitamin D deficiency disproportionately affected women and adults > 45 years old. Conclusions: Vitamin D deficiency is endemic in Saudi Arabia and strongly linked to worsened metabolic markers. Optimizing vitamin D status through screening and correcting the deficiency may provide a cost-effective approach to confronting the regional diabetes epidemic and reducing cardiovascular disease risk.

## 1. Introduction

Vitamin D is a fat-soluble vitamin that plays an essential role in bone health and calcium homeostasis [1]. In recent years, vitamin D has been demonstrated to have a much broader range of physiological functions beyond bone health, including possible roles in glucose metabolism, cardiovascular health, immune function, and cancer prevention [2]. A growing body of evidence from observational and experimental studies suggests that vitamin D deficiency may be an under-recognized risk factor for a variety of chronic diseases, including type 2 diabetes, hypertension, dyslipidemia, and cardiovascular disease [3].

The most biologically active form of vitamin D is 1,25-dihydroxyvitamin D, also known as calcitriol [4]. This active vitamin D metabolite functions similarly to a hormone by binding to the vitamin D receptor (VDR), which then interacts with DNA sequences throughout the genome to modulate the expression of a multitude of genes [5]. Through these genomic mechanisms, vitamin D signaling regulates numerous biological processes related to glucose metabolism and lipid homeostasis [6]. Given vitamin D’s pleiotropic effects mediated by VDR activation, research has focused on elucidating the potential metabolic benefits of optimizing vitamin D status [7]. However, interventional studies on vitamin D supplementation in humans have yielded mixed results, highlighting the need for further research to clarify the nature of these complex relationships [8].

Several lines of evidence indicate that vitamin D deficiency is highly prevalent worldwide and represents a critical public health problem [9]. Approximately one billion people globally are estimated to have deficient or insufficient levels of vitamin D [10]. Deficiency rates are markedly higher in at-risk groups, including older adults, patients in psychiatric institutions, individuals with obesity, and ethnic minorities with darker skin pigmentation residing at higher latitudes [11,12]. The high worldwide prevalence of vitamin D deficiency may be largely attributable to lifestyle changes in modern society, including increased time spent indoors and sun avoidance behaviors [13]. Dietary sources alone are typically not adequate to meet bodily vitamin D requirements, and, thus, regular sun exposure is essential for endogenous vitamin D synthesis in the skin [14,15]. However, concerns over skin cancer risks have led public health authorities in many countries to recommend sun avoidance and the liberal use of sunscreen. Although these guidelines aim to prevent skin cancer, they have likely contributed to endemic vitamin D deficiency at the population level [16].

Definitions of optimal vitamin D status are debated, but most experts define deficiency as 25-hydroxyvitamin D (25(OH)D) levels below 20 ng/mL (50 nmol/L); insufficiency as 25(OH)D levels between 20–29 ng/mL; and sufficiency as 25(OH)D levels greater than or equal to 30 ng/mL [17]. According to these criteria, approximately one-third to one-half of otherwise healthy adults in the United States and Europe are vitamin D-deficient [18]. These widespread suboptimal vitamin D levels underscore the potential value of vitamin D supplementation or food fortification programs as safe, low-cost interventions that could have significant public health impacts [19]. However, the lack of consensus around target 25(OH)D levels reflects ongoing uncertainties about vitamin D’s health effects and optimal dosing regimens.

The global diabetes epidemic has intensified interest in identifying modifiable risk factors for type 2 diabetes. Experimental and observational data indicate that vitamin D signaling is involved in insulin secretion and sensitivity, suggesting that vitamin D deficiency could potentially aggravate diabetes risk [20,21]. Pancreatic β-cells express VDRs and may convert circulating 25(OH)D to active 1,25(OH)2D locally, where it can exert autocrine and paracrine effects on insulin synthesis and secretion [20]. In various animal knock-out models, the targeted disruption of VDRs or vitamin D-activating enzymes results in impaired insulin secretion and glucose intolerance [22]. At the molecular level, vitamin D metabolites appear to stimulate insulin secretion by regulating calcium fluxes in β-cells. Vitamin D may also exert indirect effects by modulating extracellular calcium and calcium-regulating hormones [23].

In humans, low vitamin D levels have been linked to impaired glucose tolerance and insulin resistance in multiple cross-sectional reports. For example, Han et al. (2017) reported a significant inverse correlation between serum 25(OH)D concentrations and insulin resistance as measured via homeostatic model assessment for insulin resistance (HOMA-IR) in a sample of 126 healthy patients of Asian ethnicities [24]. Similarly, higher vitamin D levels were associated with enhanced insulin sensitivity in 381 older adults participating in the Health, Aging, and Body Composition Study [25]. However, conflicting results were reported in the National Health and Nutrition Examination Survey (NHANES) III, with no relationships observed between 25(OH)D and diabetes risk markers after adjustment for potential confounders in over 6000 adults [26].

While these observational findings are intriguing, they are limited by the possibilities of reverse causation or residual confounding from overlooked covariates [27]. Intervention studies are needed to determine whether vitamin D supplementation can directly improve glucose metabolism or prevent diabetes [28]. However, the results from the few small trials conducted to date have been mixed. Improvement in insulin sensitivity was noted with short-term high-dose ergocalciferol supplementation in 91 South Asians in New Zealand [29]. By contrast, vitamin D had no discernable effects on insulin secretion, sensitivity, or glucose in a 6-month trial involving 92 glucose-tolerant patients [30]. The available clinical data are further complicated by inconsistencies in dosing regimens, the duration of therapy, study populations, and methodological quality across trials [31]. Overall, mechanistic links between vitamin D, β-cell function, and insulin action are biologically plausible but not firmly established in humans.

## 2. Materials and Methods

### 2.1. Study Design and Participants

This retrospective, observational, cross-sectional study analyzed 476 Saudi patients (294 men and 182 women) aged 15–78 years, attending outpatient clinics at King Faisal University, Saudi Arabia, from August to November 2023. From an initial screening of 936 subjects, 476 were selected based on inclusion and exclusion criteria. The inclusion criteria were Saudi nationality, aged 15–78, and complete medical records with necessary lab tests (vitamin D, glucose, HbA1c, lipid profile). Exclusion criteria included pregnancy/lactation, conditions or medications affecting glucose/lipid metabolism, and the use of vitamin D/calcium supplements. The participants were divided into three age groups: young adults (15–30 years), middle-aged adults (31–50 years), and older adults (51–78 years). This categorization allows for an in-depth examination of vitamin D’s impact on diabetes prevalence among varied age demographics, enhancing the study’s accuracy and relevance to public health strategies.

### 2.2. Data Collection

Demographic and clinical data were extracted from electronic medical records, including serum 25(OH)D levels, fasting plasma glucose, HbA1c, and lipid profiles. A streamlined process was employed to ensure efficient data collection and quality control, including independent verification of a subset of data entries and anonymization of patient identifiers before analysis.

### 2.3. Diabetes Diagnosis Clarification

Diabetes was diagnosed based on HbA1c ≥ 6.5%, fasting glucose ≥ 126 mg/dL, or use of anti-diabetic medication, considering multiple assessments to confirm the diagnosis. Furthermore, we recognized the importance of distinguishing between type 1 and type 2 diabetes for the purposes of our study. As such, diagnoses were not only based on glycemia and HbA1c levels but also took into consideration the clinical context, including symptoms, onset age, and dependence on insulin or other medications. This differentiation was crucial for accurately categorizing participants and analyzing the relationship between vitamin D levels and diabetes type.

### 2.4. Lipid Profile Categorization

Cholesterol levels were categorized based on the following clinical guidelines: total cholesterol ≥ 240 mg/dL (elevated), LDL cholesterol ≥ 160 mg/dL (high), HDL cholesterol < 40 mg/dL in men and <50 mg/dL in women (low), and triglycerides ≥ 200 mg/dL (hypertriglyceridemia). These intervals are based on established cardiovascular risk stratification.

### 2.5. Data Analysis

Statistical analyses were performed with SPSS software version 23 (IBM, Armonk, NY, USA). According to the sample size, this study was sufficiently powered (95% power) to detect significant associations between vitamin D status groups and glucose/lipid categories. Categorical data are reported as frequencies and percentages. Cross-tabulation and Pearson’s chi-square tests were used to evaluate the relationships between vitamin D levels (deficient/insufficient/normal) and glucose and lipid profile categories. Statistical significance was defined as *p* < 0.05.

### 2.6. Ethical Considerations

The study protocol was approved by the Institutional Ethics Review Board of King Faisal University. To protect patient privacy, medical record data were anonymized by assigning coded numbers with no personal identifiers. Safeguards were implemented to ensure the confidentiality of protected health data. The study was conducted in accordance with the Declaration of Helsinki.

## 3. Results

The demographic data from Table 1 of our study provide intriguing insights into the relationship between vitamin D status and different demographic groups.

Firstly, gender differences are notable. In Group 1 (normal vitamin D levels) and Group 2 (insufficient vitamin D), males are more prevalent (26.3% and 21%, respectively) compared to females (9.0% and 13.7%, respectively). This trend reverses in Group 3 (deficient in vitamin D), where the proportion of females (15.5%) exceeds that of males (14.5%). The association between vitamin D status and gender was highly significant (*p* = 0.000013). This could suggest that men are more prone to vitamin D insufficiency than women.

Regarding age categories, the distribution suggested that vitamin D deficiency increases with age. In the 51–78 age group, the prevalence of vitamin D deficiency (18.5%) and insufficiency (18.7%) was higher than that in the younger age groups. This pattern was consistent across all vitamin D status groups; the oldest age group always had the highest prevalence of vitamin D deficiency and insufficiency. The association between age and vitamin D status was highly significant (*p* = 0.005), underscoring the increased risk of vitamin D deficiency in older populations. This could be attributed to factors such as reduced synthesis of vitamin D in the skin, dietary intake, and lifestyle changes associated with aging.

In Table 2, the data also showed a significant association between diabetes mellitus and vitamin D levels. In patients with normal vitamin D levels (Group 1), only 10.1% had diabetes, whereas this prevalence was higher in those with insufficient vitamin D levels (Group 2, 11.6%) and deficient vitamin D levels (Group 3, 18.3%). The pattern was reversed in patients without diabetes, with the highest proportion of these patients (25.2%) in Group 1 (normal vitamin D levels) and the lowest (11.8%) in Group 3 (deficient). The association between vitamin D levels and diabetes mellitus was highly significant (*p* = 0.00001). The data on fasting blood glucose further reinforced this trend. Normal glucose levels were the most prevalent in Group 1 (20.6%) and least prevalent in Group 3 (5.3%). Conversely, diabetic glucose levels (>125 mg/dL) were observed in only 10.1% of patients in Group 1, whereas a striking 18.3% of patients in Group 3 had diabetic glucose levels. This trend was highly significant (*p* = 0.00001). The levels of HbA1c, a marker of long-term glucose control, exhibited a similar association with vitamin D levels. Normal HbA1c levels were most common in the normal vitamin D group and least common in the deficient group. The distribution of patients with diabetic HbA1c levels (>6.4%) was significantly skewed; 10.1% of patients in Group 1 had diabetic HbA1c levels compared with 18.3% in Group 3 (*p* = 0.000).

This study also identified a strong association between vitamin D levels and various lipid panel results (Table 3). The patients were categorized into three groups according to their vitamin D status (normal, insufficient, and deficient), and highly significant differences were found between these groups (*p* = 0.000). First, as vitamin D levels decreased, the prevalence of high total cholesterol increased. Specifically, 18.9% of patients in the vitamin D-deficient group had high total cholesterol levels (≥240 mg/dL), whereas only 5.3% in the normal vitamin D group had high total cholesterol. This trend was also observed with LDL cholesterol levels; 13.2% of patients in the vitamin D-deficient group had high LDL levels (≥160 mg/dL), significantly higher than the 6.9% of patients with high LDL in the normal vitamin D group.

HDL cholesterol is often referred to as ‘good cholesterol’, and the data indicated a decrease in normal HDL levels with decreasing vitamin D levels; this trend was especially notable in women. Only 5.9% of women with vitamin D deficiency had normal HDL levels (≥50 mg/dL), whereas 7.8% of women in the normal vitamin D group had normal HDL levels. We observed a similar pattern for triglyceride levels. A significantly higher percentage of patients in the vitamin D-deficient group (16.0%) had high triglyceride levels (≥200 mg/dL) compared with those with normal vitamin D levels (5.5%).

## 4. Discussion

This cross-sectional analysis of 476 Saudi patients provides compelling evidence of an association between vitamin D deficiency and markers for metabolic diseases, including diabetes and dyslipidemia. We observed a significantly higher prevalence of diabetes in patients with vitamin D deficiency versus those with normal vitamin D levels (18.3% vs. 10.1%), along with significant associations between lipid abnormalities and deficient vitamin D levels; these findings have important clinical and public health implications.

### 4.1. Vitamin D and Diabetes

Notably, we demonstrated that vitamin D status was strongly correlated with diabetes prevalence. Only 10.1% of participants with sufficient vitamin D had diabetes, whereas 11.6% and 18.3% of participants in the insufficient and deficient groups had diabetes, respectively. This dose–response relationship was strikingly consistent across diagnostic criteria, including fasting glucose, HbA1c levels, and medical history. The sharply elevated proportion of patients with diabetes in the vitamin D deficiency group supported our hypothesis regarding the role of vitamin D in glucose metabolism. Mechanistic links have been established between vitamin D, insulin secretion, insulin sensitivity, and inflammation, which may explain the correlation between vitamin D status and diabetes risk [23]. Vitamin D has been shown to stimulate insulin receptor expression and regulate calcium transport involved in insulin secretion. Pancreatic β-cell VDR signaling also exerts key effects on glucose-stimulated insulin biosynthesis and release [32]. Observational data have linked low 25(OH)D concentrations to insulin resistance, impaired β-cell function, systemic inflammation, and diabetes risk [33,34]. Randomized trials are still needed, but our powerful population-level findings support the results of a previous study [35] reporting that vitamin D deficiency is a clinically relevant predictor of diabetes in the Saudi population, even after adjusting for age, sex, BMI, and socioeconomic status.

Our data indicated that 18.3% of participants in the vitamin D-deficient group had diabetes; this proportion is markedly higher than the rates reported previously, even in studies focusing on patients with diabetes. For example, a 2021 meta-analysis found an overall diabetes prevalence of 12.7% in the Kingdom of Saudi Arabia [36]. Although differences in screening methodologies must be considered, the higher rate of diabetes among participants with vitamin D deficiency strongly supports low vitamin D as a risk factor for diabetes. This has significant public health implications, given that the rate of hypovitaminosis D in Saudi Arabia is among the highest rates globally; a previous systematic review found that the mean serum 25(OH)D level in Saudi adults was only 19 ng/mL [37].

### 4.2. Vitamin D and Dyslipidemia

Our lipid profile analysis also identified vitamin D deficiency as a potential contributor to dyslipidemia. We discovered significant associations between lower 25(OH)D levels and less favorable lipid panel results, including total cholesterol, LDL cholesterol, HDL cholesterol (in women), and triglycerides. The prevalence of high total cholesterol (≥240 mg/dL) was over threefold higher in the deficient group (18.9%) than in the sufficient group (5.3%). Although research remains limited on the vitamin D–dyslipidemia relationship, one of the proposed mechanisms is the regulation of vitamin D by various lipid metabolism genes involved in de novo cholesterol synthesis [38]. Low 25(OH)D levels have also been associated with higher levels of systemic inflammation, which can independently worsen blood lipids [39,40]. These data provide preliminary population-level evidence in a Saudi cohort that screening for and correcting vitamin D deficiency may be a valuable tool to prevent and manage dyslipidemia. Cost-effectiveness studies should investigate whether improving vitamin D status could reduce cumulative pharmaceutical spending from expanded statin prescriptions. At a minimum, testing 25(OH)D levels could serve as an inexpensive biomarker for identifying high-risk patients who require aggressive lipid modulation.

### 4.3. Demographic Risk Factors

Our stratified analysis also revealed disproportionately high rates of vitamin D insufficiency among women (29.2%) and adults over 45 years old (37.2%); these findings are consistent with those of regional Middle Eastern reports. Cultural dress norms in Saudi Arabia likely limit sunlight-dependent vitamin D synthesis in women due to increased skin coverage and avoidance of public outdoor spaces [41]. Older individuals may also spend more time indoors, which could contribute to depleted vitamin D reserves. Our findings confirming age and sex as risk factors suggest that female gender and older age (over 45 years) could act as indicators for more intensive screening and supplementation approaches to efficiently direct resources to the most vulnerable groups [42].

From a health systems perspective, the worryingly endemic vitamin D deficiency observed calls into question the appropriateness of the 30 ng/mL 25(OH)D threshold for defining sufficiency. Mounting longitudinal evidence links sustained values between 30–40 ng/mL with better health outcomes compared with marginally sufficient levels, suggesting that a higher cutoff for Saudi Arabia may be warranted [43]. Implementing such a cutoff would significantly expand the proportion of the population categorized as vitamin D-deficient, communicating a stronger urgency for nationwide education and food fortification policies targeting long-term maintenance in the 40+ ng/mL range.

Our study observed a gender distribution of 294 men and 182 women among the participants. This distribution is in alignment with existing epidemiological data for our region, which demonstrates a higher prevalence of vitamin D deficiency among women. To contextualize our findings within the broader scientific literature, we have referenced several epidemiological studies that support this gender-related pattern in vitamin D deficiency prevalence.

We acknowledge the potential for bias introduced by this gender distribution in our analysis. The disproportionate representation of genders could influence this study’s outcomes, particularly in understanding the correlation between vitamin D levels and metabolic markers. To address this, we have conducted a thorough examination of the impact this distribution may have on our results. Our analysis suggests that, while gender distribution reflects regional health trends, it is crucial to consider these demographic differences when interpreting this study’s findings [44,45].

Further research is encouraged to explore the underlying causes of this gender disparity in vitamin D deficiency and its implications for metabolic health. By acknowledging and discussing these aspects, we aim to contribute to a more nuanced understanding of vitamin D’s role in metabolic syndrome, emphasizing the need for gender-specific approaches in prevention and treatment strategies.

## 5. Limitations and Future Research

One limitation of this study is its cross-sectional design, which restricts causal inference between vitamin D status and metabolic markers. Although the associations were highly probable, given the significant *p*-values, prospective cohort analyses are required to confirm that vitamin D deficiency precedes dysglycemia and dyslipidemia and does not result from reverse causation. Multi-year studies tracking participants’ vitamin D levels over regular intervals could establish temporality regarding the development of metabolic abnormalities. Large-scale randomized controlled trials designed specifically to test the effects of correcting vitamin D deficiency on glycemic and lipid profiles would provide even more robust evidence. Demonstrating the reversibility of metabolic risk factors with vitamin D replenishment would conclusively verify low vitamin D status as a modifiable determinant of diabetes and cardiovascular disease development.

Another limitation is that residual confounding from unmeasured variables may have influenced the observed associations. We adjusted for likely demographic and lifestyle confounders, but factors such as exercise, sun exposure behaviors, clothing coverage, nutrition, alcohol use, and sleep patterns could still explain some differences between groups. Additionally, the convenience sampling strategy may have restricted the generalizability of the prevalence and incidence. Population-based cohort studies with probability sampling would achieve a superior representation of the Saudi adult population. Nevertheless, the highly significant *p*-values indicate that substantial demographic confounding is unlikely.

In conclusion, addressing the high burden of vitamin D deficiency in Saudi Arabia appears to be warranted, given its links to an increased risk of metabolic disease. Optimizing vitamin D status through food fortification policies targeting the 40–50 ng/mL range for 25(OH)D could significantly contribute to efforts confronting the escalating regional diabetes epidemic. Future progress will depend on prioritizing funding for masked hypovitaminosis D research and increasing public awareness that vitamin D screening and sensible sunlight exposure may unlock immense potential for preventing chronic disease.

## Figures and Tables

**Table 1:** Demographic data of the patients in this study.

	Group 1 Normal Vitamin D Levels	Group 2 Vitamin D Insufficiency	Group 3 Vitamin D Deficiency	*p*-Value
Gender				
Male	125 (26.3%)	100 (21.0%)	69 (14.5%)	0.000013
Female	43 (9.0%)	65 (13.7%)	74 (15.5%)	
Age category				
15–30	9 (1.9%)	22 (4.6%)	22 (4.6%)	0.005
31–50	41 (8.6%)	54 (11.3%)	33 (6.9%)	
51–78	118 (24.8%)	89 (18.7%)	88 (18.5%)	

**Table 2:** Association between vitamin D levels and the diabetes status of the patients.

	Group 1 Normal Vitamin D Levels	Group 2 Vitamin D Insufficiency	Group 3 Vitamin D Deficiency	*p*-Value
Diabetic mellitus status				0.00001
Diabetic	48 (10.1%)	55 (11.6%)	87 (18.3%)	
Non-diabetic	120 (25.2%)	110 (23.1%)	56 (11.8%)	
Fasting blood glucose level (mg/dL)				0.00001
Normal (70–99 mg/dL)	98 (20.6%)	84 (17.6%)	25 (5.3%)	
Pre-diabetic (100–125 mg/dL)	22 (4.6%)	26 (5.5%)	31 (6.5%)	
Diabetic (≥126 mg/dL)	48 (10.1%)	55 (11.6%)	87 (18.3%)	
HgA1c level (%)				0.000
Normal (<5.7%)	99 (20.8%)	83 (17.4%)	25 (5.3%)	
Pre-diabetic (5.7–6.4%)	21 (4.4%)	27 (5.7%)	31 (6.5%)	
Diabetic (≥6.5%)	48 (10.1%)	55 (11.6%)	87 (18.3%)	

**Table 3:** Association between vitamin D levels and the lipid profile among the patients.

Lipid Profile Category		Group 1: Normal Vitamin D Levels	Group 2: Vitamin D Insufficiency	Group 3: Vitamin D Deficiency	*p*-Value	*X*^2^ Statistic
**Total Cholesterol Level (mg/dL)**	Normal (<200 mg/dL)	110 (23.1%)	57 (12.0%)	13 (2.7%)	0.000	114.74
	Borderline (200–239 mg/dL)	33 (6.9%)	39 (8.2%)	40 (8.4%)		
	High (≥240 mg/dL)	25 (5.3%)	69 (14.5%)	90 (18.9%)		
**LDL Level (mg/dL)**	Optimal (<100 mg/dL)	77 (16.2%)	61 (12.8%)	28 (5.9%)	0.000	31.68
	Borderline (100–159 mg/dL)	58 (12.2%)	59 (12.4%)	52 (10.9%)		
	High (≥160 mg/dL)	33 (6.9%)	45 (9.5%)	63 (13.2%)		
**HDL Level (mg/dL)**	Normal (Male) (≥40 mg/dL)	83 (17.4%)	59 (12.4%)	23 (4.8%)	0.000	21.49
	Normal (Female) (≥50 mg/dL)	37 (7.8%)	39 (8.2%)	28 (5.9%)		
	Low (Male) (<40 mg/dL)	42 (8.8%)	41 (8.6%)	46 (9.7%)		
	Low (Female) (<50 mg/dL)	6 (1.3%)	26 (5.5%)	46 (9.7%)		
**Triglyceride Level (mg/dL)**	Normal (<150 mg/dL)	105 (22.1%)	53 (11.1%)	30 (6.3%)	0.000	83.13
	Borderline (150–199 mg/dL)	37 (7.8%)	62 (13.0%)	37 (7.8%)		
	High level (≥200 mg/dL)	26 (5.5%)	50 (10.5%)	76 (16.0%)		

## Data Availability

The data presented in this study are available on request from the corresponding author.
